# Association of eNOS Gene Polymorphisms G894T and T-786C with Risk of Hepatorenal Syndrome

**DOI:** 10.1155/2016/2579626

**Published:** 2016-08-10

**Authors:** Yuksel Seckin, Ali Yigit, Elif Yesilada, Gonca Gulbay, Yasir Furkan Cagin, Harika Gozukara, Yılmaz Bılgıc, Oguzhan Yildirim, Yusuf Turkoz, Zeynep Aksungur

**Affiliations:** ^1^Department of Gastroenterology, Faculty of Medicine, Inonu University, 44280 Malatya, Turkey; ^2^Department of Internal Medicine, Faculty of Medicine, Inonu University, 44280 Malatya, Turkey; ^3^Department of Medical Biology and Genetics, Faculty of Medicine, Inonu University, 44280 Malatya, Turkey; ^4^Department of Biostatistics, Faculty of Medicine, Inonu University, 44280 Malatya, Turkey; ^5^Department of Biochemistry, Faculty of Medicine, Inonu University, 44280 Malatya, Turkey

## Abstract

*Background*. There are no studies investigating the relationship between endothelial nitric oxide synthase (eNOS) gene polymorphisms and hepatorenal syndrome (HRS).* Aim.* The purpose of this study is to elucidate whether eNOS gene polymorphisms (G894T and T-786C) play a role in the development of type-2 HRS.* Methods*. This study was carried out in a group of 92 patients with cirrhosis (44 patients with type-2 HRS and 48 without HRS) and 50 healthy controls. Polymorphisms were determined by polymerase chain reaction (PCR) and melting curve analysis.* Results*. We did not find any significant difference in allele and genotype distributions of the eNOS -T-786C polymorphism among the groups (*p* = 0.440). However, the frequency of GT (40.9%) and TT (13.6%) genotypes and mutant allele T (34.1%) for the eNOS G894T polymorphism were significantly higher (*p* < 0.001 and *p* < 0.001, resp.) in the HRS group than in both the stable cirrhosis (14.6%, 4.2%, and 11.5%, resp.) and the control (22.0%, 2.0%, and 13.0%, resp.) groups.* Conclusion*. The occurrence of mutant genotypes (GT/TT) and mutant allele T in eNOS -G894T polymorphisms should be considered as a potential risk factor in cirrhotic patients with HRS.

## 1. Introduction

Hepatorenal syndrome (HRS), one of the most severe complications of cirrhosis, is defined as functional renal failure in the course of an acute liver injury and/or chronic liver disease. Two types of HRS in cirrhosis were initially defined by the International Ascites Club based on major and minor criteria. Type-1 HRS is a form of rapidly progressing renal failure defined by a doubling of the baseline serum creatinine to a level greater than 2.5 mg/dL in less than two weeks. Type-2 HRS is characterized by a steady or slow increase in serum creatinine levels to over 1.5 mg/dL. Type-2 HRS is frequently associated with refractory ascites, whereas type-1 HRS is usually triggered by infection. Cases of untreated type-1 HRS have a remarkably low survival rate in comparison to untreated cases of type-2 HRS [1, 2].

The pathogenetic pathway underlying the development of HRS is not completely understood. HRS develops as a result of abnormal hemodynamics, leading to splanchnic and systemic vasodilatation along with renal vasoconstriction [3]. Nitric oxide (NO) is a potent vasodilator that plays an important role in this abnormal hemodynamics. Some studies have suggested that NO is elevated in the peripheral circulation of patients with cirrhosis [4, 5].

NO is produced from L-arginine by three nitric oxide synthase (NOS) isoenzymes: neuronal nitric oxide synthase (nNOS), inducible nitric oxide synthase (iNOS), and endothelial nitric oxide synthase (eNOS). The human eNOS gene is located on chromosome 7 and contains 26 exons. There are many functional polymorphisms in different regions of the eNOS gene [6]. A functional polymorphism in exon 7 of human eNOS corresponds to a Glu-Asp change at codon 298 (Glu298Asp, G894T) (*rs1799983*). eNOS G894T allele carriers tend to exhibit diminished eNOS enzyme activity in comparison to GG homozygotes [7]. Second functional polymorphism is a point mutation of thymine to cytosine at nucleotide -786 (T-786C) (*rs2070744*) in the 5′-flanking region of the eNOS gene, which can result in a significant decrease in promoter activity [8] and significantly reduce serum NO levels [9].

Although many studies have investigated NO levels in cirrhotic patients, the present study aimed to elucidate whether two eNOS polymorphisms (G894T and T-786C) play a role in the development of HRS.

## 2. Materials and Methods

### 2.1. Study Design

This was a prospective study, which was conducted at inpatient and outpatient hepatology clinics of the Inonu University Medical Faculty. The study was approved by the ethical committee of the institution involved, in compliance with the Declaration of Helsinki. All participants signed an informed consent document before enrolling in the study.

### 2.2. Participants

A total of 92 patients with cirrhosis (44 patients with type-2 HRS and 48 patients without HRS) and 50 controls were included in the study. Participants were classified into three study groups as follows.

*Group-1: Type-2 Hepatorenal Syndrome (HRS)*. The 44 patients recruited in this group had been diagnosed with type-2 HRS according to the criteria of the International Ascites Club [1]. These patients also had a clinical and/or histopathological diagnosis of cirrhosis. Serum creatinine levels in this group were > 1.5 mg/dL. The exclusion criteria were shock, fluid losses, concurrent treatment with nephrotoxic drugs, proteinuria of greater than 500 mg/day as measured by 24-hour urine collection, more than 50 urine red blood cells per high-power field, and patients who did not fulfill HRS diagnostic criteria. Patients with ascitic fluid infection and other active infections, patients that have been receiving nitrates, and patients with bleeding esophageal varices were not included. Abdominal and renal ultrasounds were performed to exclude a postrenal cause for renal dysfunction and to check for the presence of ascites. For patients with HRS, diuretics were interrupted for at least 48 hours. Albumin and/or saline were infused to prevent prerenal acute kidney injury as a result of volume depletion. 

*Group-2: Stable Cirrhosis*. This group included 48 patients with clinical and/or histopathological diagnosis of cirrhosis secondary to various causes, including alcohol consumption, viral hepatitis, autoimmune hepatitis, or unknown etiology. These patients had serum creatinine values < 1.5 mg/dL. Cases in this group were enrolled from patients who presented to clinics for routine procedures and check-ups. These patients had no renal dysfunction, active infection, or malignancy. 

*Group-3: Control*. Fifty adult volunteers were recruited from the hepatology outpatient clinic to serve as controls. They had no liver or renal disease, acute infection, inflammatory disease, or malignancy.

### 2.3. Demographic Data and Collection of Blood Samples

Demographic characteristics were also recorded via face-to-face patient interviews. Peripheral blood samples were collected from all participants to perform blood counts, routine biochemistry, prothrombin time (PT), and eNOS gene polymorphism molecular analysis. Participants also provided freshly voided first morning urine for urinalysis. Model for End-Stage Liver Disease (MELD) and Modification of Diet in Renal Disease (MDRD) scores were calculated for all patients included in the study. Glomerular filtration rates (GFR) were calculated using the MDRD equation.

### 2.4. eNOS Gene Polymorphism Molecular Analysis

#### 2.4.1. Isolation of Genomic DNA

Genomic DNA was isolated from peripheral blood leukocytes using High Pure PCR Template Preparation Kits (Roche Diagnostics GmbH, Mannheim, Germany) according to the manufacturer's instructions. Kits were stored at −20°C until analysis.

#### 2.4.2. Genotype Analysis

Determination of polymorphisms (G894T [GLu298Asp] [E298D] [*rs1799983*] and -786 T>C [*rs20707440*]) was performed using LightCycler real time PCR (Roche Diagnostics, Mannheim, Germany) and a LightSNiP assay from TIB MOLBIOL (Berlin, Germany). A 20 *μ*L mixed reaction solution—which contained 10.4 *μ*L of water, 2 *μ*L of LC*™* FastStart DNA Master HybProbe kit (Roche Diagnostics), 1.0 *μ*L of LightSNiP reagent mix, 1.6 *μ*L MgCl_2_ (25 mM), and 5 *μ*L of genomic DNA—was transferred into capillary tubes. PCR conditions were as follows: 10 minutes for initial denaturation at 95°C, 45 cycles at 95°C for 10 seconds for further denaturation, 10 seconds at 60°C for annealing, and 15 seconds at 72°C for extension. The melting point analysis uses fluorescence resonance energy transfer to detect polymorphic sites. Melting curve analysis was performed with an initial denaturation at 9°C for 20 seconds, 20 seconds of denaturation at 40°C, slow heating to 85°C with a ramping rate of 0.2°C/second, and continuous fluorescence detection. Polymorphic, mutated, and wild-type alleles were identified by the specific melting temperature (*T*_*m*_) of the resulting amplicons. *T*_*m*_ values for the G894T (*rs1799983*) G and T alleles were 60.01°C and 65.98°C, respectively, and for the -786 T>C (*rs2070744*) C and T alleles were 59.09°C and 66.17°C, respectively. The values for the respective melting temperatures varied by ±2.5°C between different experiments. Genotype screening was performed simultaneously for cases and controls.

#### 2.4.3. Plasma Nitrite Assay

Plasma nitrite levels were measured as total nitrite with the spectrophotometric Griess reaction. This was done because it has been shown that plasma nitrite/nitrate is an index of endogenous NO production [10, 11]. The procedure was partly adapted from the method described by Ozbek et al. [12]. First, all plasma samples were deproteinized. Briefly, for every 200 *µ*L plasma sample, 400 *µ*L of 10% zinc sulfate and 400 *µ*L of 0.5 N sodium hydroxide were added. The plasma samples were then vortexed and centrifuged at 25.000 ×g for five minutes at 4°C.

For this study, 200 *µ*L of deproteinized aliquot or water blank was incubated in a final volume of 750 *µ*L containing 75 *µ*L of 0.32 mol/L potassium phosphate buffer (pH 7.5), 25 *µ*L of nitrate reductase (Sigma) with NADPH (50 *µ*mol/L) and FAD (5 *µ*mol/L) as coenzymes, and 650 *µ*L of water for two hours. After reducing nitrate to nitrite with nitrate reductase, total nitrite was determined with Griess reagent by mixing equal volumes of the reduced samples with Griess reagent (a 1 : 1 ratio of 0.1%  *α*-naphthylamine in water/1% p-aminobenzene sulfamide in 5% phosphoric acid). The samples were allowed to stand for 15 minutes and then read in a spectrophotometer at an absorbance of 548 nm. A range of sodium nitrite standards (0–100 *µ*mol/L) was prepared, and a standard curve was used to convert plasma sample measurements to micromoles per liter of nitrite. The reaction was linear from 0.25 to 100 *µ*mol/L. Assays were performed as duplicates. The interassay and intra-assay coefficients of variation for the assay were < 7% and < 4%, respectively.

### 2.5. Statistical Methods

Normally distributed data were summarized by mean and standard deviation. Comparison of the groups in terms of these variables was performed by one-way ANOVA, and the Tukey method was used for pairwise comparisons. For nonnormally distributed data, median, minimum, and maximum values were used to express the data, and the Kruskal-Wallis test was applied for comparison of the groups. After the Kruskal-Wallis test, the Conover method was used for multiple comparisons. Categorical data are presented in terms of count and percentage. Chi-square tests were used to test genotypic association and allelic association and to test for the presence of the Hardy-Weinberg equilibrium. Multinomial logistic regression analyses were used for odds ratio estimations. Power analysis was done to provide reliable and realistic results of our study. Power values of statistical analysis for genotype frequencies were calculated as 95.4% to 93.1% allele frequency. In all analyses, the significance level was considered to be 0.05. The Statistical Package for the Social Sciences (SPSS ver. 21.0 for Windows; SPSS, Chicago, IL, USA) was used for all analyses.

## 3. Results

A total of 92 patients with cirrhosis (44 patients with type-2 HRS and 48 without HRS) and 50 controls were included in this study. The patients with HRS included 16 (36.4%) women and 28 (63.6%) men. The patients with stable cirrhosis included 17 (35.4%) women and 31 (64.6%) men. There were no differences between the control group and the other groups in terms of age or sex. However, patients in the stable cirrhosis group were significantly younger than those in the HRS group (*p* < 0.05).

Underlying etiologies for cirrhosis included hepatitis B in 44 (47.8%) patients, hepatitis C in 16 (17.4%) patients, autoimmune hepatitis in six (6.5%) patients, alcoholic liver disease in 17 (18.5%) patients, and cryptogenic cirrhosis in nine (9.8%) patients. Patients with HRS had significantly higher average MELD scores (*p* < 0.001) compared with those in the stable cirrhosis group (27 versus 13). GFR (30.5–90.99) was lower in the HRS group than in the other groups. Ascites were found in all patients with HRS. The clinical characteristics of the study groups are presented in Table 1.

### 3.1. Endothelial Nitric Oxide Synthase G894T Polymorphism

The frequency of GT (40.9%) and TT (13.6%) genotypes and mutant allele T (34.1%) for the eNOS G894T polymorphism were significantly higher (*p* < 0.001) in the HRS group than in the stable cirrhosis group (14.6%, 4.2%, and 11.5%, resp.). The results of this distribution are presented in Tables 2 and 3.

We compared GG, GT, and TT genotype frequencies and assessed risk ratios according to genotype among the groups in various combinations. The risk of development of HRS in HRS patients with the GT genotype was 5 times higher (OR = 5.014, 95% CI = 1.797–13.990) than in stable cirrhosis patients with the GG genotype. However, the risk of development of HRS increased 3.1-fold (OR = 3.109, 95% CI = 1.233–7.841) in HRS patients with the GT genotype in comparison to patients in the control group with the GG genotype.

The risk of development of HRS increased by 5.8 (OR = 5.850, 95% CI = 1.081–31.661) and 11.4-fold (OR = 11.400, 95% CI = 1.282–101.368) in patients with the G894 TT genotype of HRS in comparison to the stable cirrhosis and control groups with the GG genotype, respectively (Table 2). There was no statistically significant difference in terms of genotype distribution between the cirrhosis group and controls (Table 2).

Allelic frequencies were compared in order to assess risk ratios based on type of allele among the groups. There was a significant difference between the distribution of T alleles in the HRS group and that of the stable cirrhosis group, with the HRS group exhibiting a 3.4-fold (OR = 3.462, 95% CI = 1.667–7.188) increased risk of HRS. There was also a significant difference in the distribution of T alleles between the HRS group and the control group, with the HRS group exhibiting a 3.9-fold (OR = 3.997, 95% CI = 1.856–8.609) increased risk of HRS.

There were no statistically significant differences in the association of genotype and allele frequencies of eNOS 894G/T gene polymorphisms between the stable cirrhosis and the control groups (*p* > 0.05) (Tables 2 and 3, resp.).

### 3.2. Endothelial Nitric Oxide Synthase -786T/C Polymorphism

There were no significant differences in genotype and allele distributions of the eNOS 786T/C polymorphism among the HRS, stable cirrhosis, and control groups (*p* > 0.05) (Tables 2 and 3).

### 3.3. Plasma Nitrite Levels

Nitrite levels in the HRS group were significantly higher than in the stable cirrhosis and control groups (*p* < 0.001 and *p* < 0.001, resp.). Nitrite levels in patients with eNOS G894T homozygous TT genotype were significantly lower compared to those in patients with GG or GT genotypes in the HRS group (*p* < 0.001 and *p* < 0.001, resp.). The plasma nitrite levels are presented in Table 4.

## 4. Discussion

In this study, we determined that eNOS polymorphism for G894T is a risk factor in the development of HRS, despite finding no relationship between the eNOS polymorphism for T-786C and HRS. This experiment is the first study on the relationship between eNOS gene polymorphisms and HRS.

Renal vasoconstriction triggered by splanchnic and systemic vasodilatation is the most important mechanism in the development of HRS. NO is a potent vasodilator that plays a key role in these abnormal hemodynamics. L-Arginine (L-Arg), a nonessential amino acid, is the precursor for NO production by nitric oxide synthase (NOS) [13]. In patients with cirrhosis, hepatic clearance of amino acids, including L-Arginine, is decreased, and this may partially explain increased NO due to increased levels of substrate [14, 15]. NO has a very short half-life; therefore, measurements of the NO metabolites, nitrite and nitrate, are commonly used to estimate plasma NO levels. The concentration of nitrite rather than nitrate is a better marker for detection of endogenous NO concentration. In this study, the concentrations of nitrite were assessed, not NO concentration.

It has been shown that NO levels are increased in cirrhotic patients, especially in the decompensated subgroup; yet, a clear association between NO levels and renal dysfunction has not been demonstrated [4, 5]. In a study conducted by Kayali et al., NO levels were found to be increased in cirrhotic patients with renal dysfunction, and the highest levels were observed in patients with HRS. One of the notable findings in this study was the fact that L-arginine levels were increased in these patients [16].

There are few studies on the effect of NO on renal hemodynamics. Although La Villa et al. observed that renal plasma flow increased in patients with compensated cirrhosis whose NO levels were inhibited by N(G)-monomethyl-L-arginine (L-NMMA), Thiesson et al. detected a reduction in the renal plasma flow of patients with decompensated cirrhosis [17, 18]. However, another randomized placebo-control study found that renal plasma flow decreased as a consequence of inhibition of NO, and NO is an important renal vasodilator and natriuretic in patients with cirrhosis [19].

In recent years, the number of studies investigating the relationship between eNOS gene polymorphisms and NO has gradually increased. In a study conducted with healthy men, no significant association was found between eNOS genotypes and NO levels, whereas a significant increase was found in the frequency of alleles C (promoter), 4b (intron 4), and Glu (exon 7) in the group with low NO levels [20]. However, in healthy African-American subjects, it was found that T-786C and Glu298Asp polymorphisms did not affect production of NO, although the frequency of the “C-4b-Glu” haplotype was significantly increased in patients with low NO levels [21]. Tsukada et al. found that plasma NO levels of subjects who are homozygous for the a allele were nearly 20% lower than those in subjects with the b allele [22]. Another study emphasized that different eNOS gene polymorphism (eNOS tag SNP) by alternating nitrite concentration could affect cardiovascular health of children and adolescents [23].

Studies investigating the relationship between eNOS gene polymorphisms and coronary heart disease are especially notable. eNOS T-786C polymorphism has been observed to be associated with decreased NO production and coronary artery spasm [9], and eNOS T-786C mutation in white Americans with Prinzmetal's variant angina pectoris (PVA) has been identified as a reversible etiological factor. It has been confirmed that administration of L-arginine, a NO precursor, to these subjects improves PVA symptoms [24]. In a meta-analysis conducted by Luo et al., it was emphasized that eNOS G894T polymorphism was a leading factor in increased myocardial infarction (MI) risk in Asians, and ethnicity was found to play a significant role in the development of MI [25]. In the light of these studies, ethnicity has a significant impact on NO concentrations and other different eNOS gene polymorphisms (not only eNOS G894T and T-786C) [23]. Therefore, participants who were from different regions and various ethnic groups were not included.

On the basis of the above studies, it is observed that eNOS gene polymorphism results in low NO production, which triggers vasoconstriction. However, splenic vasodilation develops primarily, and renal vasoconstriction subsequently establishes in the pathogenesis of HRS with high NO levels. This study demonstrates that the eNOS G894T homozygote mutant (TT) and heterozygote (GT) genotypes are associated with significantly increased risk of developing HRS. In the HRS group, particularly in subjects with the eNOS G894T homozygous TT genotype, the significantly low nitrite levels were remarkable. These results confirm that eNOS polymorphism causes a decline in the production of NO.

In conclusion, this study found that eNOS gene polymorphism is a risk factor for HRS development. Large-scale prospective studies, including concurrent assessment of serum NO levels, are needed to fully elucidate the effects of eNOS polymorphism (especially G894T) on susceptibility to HRS.

## Figures and Tables

**Table 1:** Mean age and laboratory data of the study groups.

Parameters	HRS group (*n* = 44)	Stable cirrhosis group (*n* = 48)	Control subjects (*n* = 50)	*p* value
Age (years)^*∗*^	56.76 ± 11.66	49.06 ± 13.05^c^	51.02 ± 10.24	<0.05
AST (U/L)^*∗*^	141.85 ± 32.57^a,b^	56.88 ± 9.03^a,c^	26.88 ± 3.65^b,c^	<0.001
ALT (U/L)^*∗*^	87.69 ± 20.93^a,b^	52.17 ± 8.82^a,c^	30.24 ± 2.93^b,c^	<0.001
Albumin (g/dL)^*∗*^	2.47 ± 0.18^a,b^	3.22 ± 0.14^a,c^	4.23 ± 0.20^b,c^	<0.001
INR^*∗*^	1.79 ± 0.19^a,b^	1.27 ± 0.17^a,c^	0.93 ± 0.05^b,c^	<0.001
Total bilirubin (mg/dL)^*∗*^	6.57 ± 1.96^a,b^	2.84 ± 0.81^a,c^	0.84 ± 0.08^b,c^	<0.001
eGFR (mL/min/1.73 m^2^)^*∗*^	31.69 ± 4.93^a,b^	90.92 ± 3.37^c^	99.18 ± 0.96^c^	<0.001
Creatinine (mg/dL)^*∗*^	2.11 ± 0.18^a,b^	0.75 ± 0.08	0.74 ± 0.12	<0.001
MELD score^*∗*^	26.35 ± 1.91^b^	13.06 ± 1.76^c^	—	<0.001

HRS, hepatorenal syndrome; AST, aspartate transaminase; ALT, alanine transaminase; eGFR, estimated glomerular filtration rate; MELD, model for end-stage liver disease.

^*∗*^Quantitative values are expressed as median ± standard deviation.

^a^ *p* < 0.05 versus control group, ^b^ *p* < 0.05 versus stable cirrhosis group, and ^c^ *p* < 0.05 versus HRS group.

**Table 2:** Genotype frequencies of *eNOS *polymorphisms 894G/T and -786T/C in HRS, cirrhosis, and control groups.

894G/T	GG		GT		TT		*χ* ^2^	*p* value	Hardy-Weinberg	
	*n*	%	*n*	%	*n*	%			*χ*^2	*p* value
HRS (*n* = 44)	20	45.5	18	40.9^a,b^	6	13.6^a,b^	17.131	<0.001	0.353	>0.05
Cirrhosis (*n* = 48)	39	81.3	7	14.6	2	4.2			3.797	>0.05
Control (*n* = 50)	38	76.0	11	22.0	1	2.0			0.037	>0.05

-786T/C	TT		TC		CC		*χ* ^2^	*p* value	Hardy-Weinberg	
	*n*	%	*n*	%	*n*	%			*χ* ^2^	*p* value

HRS (*n* = 44)	26	59.1	13	29.5	5	11.4	2.631	>0.05	2.425	>0.05
Cirrhosis (*n* = 48)	32	66.7	11	22.9	5	10.4			5.212	>0.05
Control (*n* = 50)	33	66.0	15	30.0	2	4.0			0.032	>0.05

HRS, hepatorenal syndrome; *χ* ^2^, Chi-square test.

^a^ *p* < 0.05 versus control group and ^b^ *p* < 0.05 versus cirrhosis group.

**Table 3:** Allele frequencies of *eNOS *polymorphisms 894G/T and -786T/C in HRS, cirrhosis, and control groups.

	894G/T				*χ* ^2^	*p* value	-786T/C				*χ* ^2^	*p* value
	G		T				T		C			
	*n*	%	*n*	%			*n*	%	*n*	%		
HRS (*n* = 44)	58	65.9	30	34.1^a,b^	18.898	<0.001	65	73.9	23	26.1	1.389	>0.05
Cirrhosis (*n* = 48)	85	88.5	11	11.5			75	78.1	21	21.9		
Control (*n* = 50)	87	87.0	13	13.0			81	81.0	19	19.0		

HRS, hepatorenal syndrome; *χ* ^2^, Chi-Square test.

^a^ *p* < 0.05 versus control group and ^b^ *p* < 0.05 versus cirrhosis group.

**Table 4:** Plasma nitrite levels (*μ*mol/L) in HRS, cirrhosis, and control groups.

Groups	Totally median (Min–Max)	*p*	G894T median (Min–Max)				T/C 786 median (Min–Max)			
			GG	GT	TT	*p*	TT	TC	CC	*p*
HRS (*n* = 26)	39.7^a,b^ (31.7–73.6)	<0.001	48.8 (35.3–73.6)	39.5^c^ (34.3–42.7)	32.8^c,d^ (31.7–39.4)	<0.001	41.5 (34.3–73.6)	39.5 (32.7–48.8)	36 (31.7–46.2)	0.058
Cirrhosis (*n* = 24)	34.5^b^ (28.6–45.6)		35.6 (28.6–45.6)	34.5 (34.3–34.7)	30.9 (30.0–39.8)	0.780	36.1 (30.9–45.6)	33.0 (28.6–37.6)	30.7^e^ (30.0–34.7)	0.029
Control (*n* = 20)	25.4 (20.2–29.7)		25.9 (23.4–29.0)	25.1 (23.1–29.7)	20.2 (20.2–20.2)	0.307	25.4 (23.1–29.7)	25.4 (20.2–28.1)	25.3 (23.6–27.1)	0.992

HRS, hepatorenal syndrome; plasma nitrite level (*μ*mol/L).

^a^ *p* < 0.05 versus cirrhosis group, ^b^ *p* < 0.05 versus control group, ^c^ *p* < 0.05 versus HRS with G894T GG genotype, ^d^ *p* < 0.05 versus HRS with G894T GT genotype, and ^e^ *p* < 0.05 versus cirrhosis with T786C TT genotype.
